# Intestinal Parasites Coinfection Does Not Alter Plasma Cytokines Profile Elicited in Acute Malaria in Subjects from Endemic Area of Brazil

**DOI:** 10.1155/2014/857245

**Published:** 2014-09-16

**Authors:** Juan Camilo Sánchez-Arcila, Daiana de Souza Perce-da-Silva, Mariana Pinheiro Alves Vasconcelos, Rodrigo Nunes Rodrigues-da-Silva, Virginia Araujo Pereira, Cesarino Junior Lima Aprígio, Cleoni Alves Mendes Lima, Bruna de Paula Fonseca e Fonseca, Dalma Maria Banic, Josué da Costa Lima-Junior, Joseli Oliveira-Ferreira

**Affiliations:** ^1^Laboratório de Imunoparasitologia, Instituto Oswaldo Cruz, Fundação Oswaldo Cruz, 21040-900 Rio de Janeiro, RJ, Brazil; ^2^Laboratório de Simulídeos e Oncocercose, Instituto Oswaldo Cruz, Fundação Oswaldo Cruz, 21040-900 Rio de Janeiro, RJ, Brazil; ^3^Instituto de Infectologia Emílio Ribas, 01246-900 São Paulo, SP, Brazil; ^4^Agencia de Vigilância em Saúde da Secretaria de Estado da Saúde (AGEVISA), 78900-000 Porto Velho, RO, Brazil; ^5^Centro Interdepartamental de Biologia Experimental e Biotecnologia, Universidade Federal de Rondonia, 78900-000 Porto Velho, RO, Brazil; ^6^Laboratório de Tecnologia Diagnóstica, Bio-Manguinhos, Fundação Oswaldo Cruz, 21040-900 Rio de Janeiro, RJ, Brazil

## Abstract

In Brazil, malaria is prevalent in the Amazon region and these regions coincide with high prevalence of intestinal parasites but few studies explore the interaction between malaria and other parasites. Therefore, the present study evaluates changes in cytokine, chemokine, C-reactive protein, and nitric oxide (NO) concentrations in 264 individuals, comparing plasma from infected individuals with concurrent malaria and intestinal parasites to individuals with either malaria infection alone and uninfected. In the studied population 24% of the individuals were infected with *Plasmodium* and 18% coinfected with intestinal parasites. Protozoan parasites comprised the bulk of the intestinal parasites infections and subjects infected with intestinal parasites were more likely to have malaria. The use of principal component analysis and cluster analysis associated increased levels of IL-6, TNF-*α*, IL-10, and CRP and low levels of IL-17A predominantly with individuals with malaria alone and coinfected individuals. In contrast, low levels of almost all inflammatory mediators were associated predominantly with individuals uninfected while increased levels of IL-17A were associated predominantly with individuals with intestinal parasites only. In conclusion, our data suggest that, in our population, the infection with intestinal parasites (mainly protozoan) does not modify the pattern of cytokine production in individuals infected with *P. falciparum* and *P. vivax*.

## 1. Introduction

The geographic distribution of* Plasmodium* and intestinal parasites are overlapped over the world; therefore malaria coinfection with intestinal parasites is common in tropical regions of the planet [1]. Although it is well known that polyparasitism is a common condition in human populations, its real impact on the immunopathology of other diseases, including malaria, has not been fully explored. In Brazil, malaria is endemic in the Amazon region and this area coincides with high prevalence of other diseases. The prevalence of intestinal parasites has been largely reported in the Brazilian Amazon, mainly in studies involving indigenous populations or the impact of intestinal parasitism in nutritional status on Amazonian communities [2–9]. However, the interaction between malaria and other parasites has not been explored and to our knowledge only one work addressed malaria and helminthes coinfections in children infected with* P. vivax* in the Brazilian Amazon studying [10].

Studies from human populations conducted in Africa revealed that helminthic infection can have a negative effect on host response to malaria, including increased susceptibility to* Plasmodium* infection and increased severity of disease [11–14]. However, a protective effect, such as decreased risk of cerebral malaria and lower incidence of malaria, [15–17] and no effect in the susceptibility to malaria or in the pathologic effect of* Plasmodium* infections were also reported [18, 19]. The immunological interactions between helminthes and malarial parasites are unclear and there are little consensus on the effect of malaria and helminthes coinfection. During malaria infection, cytokines are reported to be important molecular markers of cell-mediated immune response and known to be critical players in the regulation of diseases. A T helper 1 (Th1) response predominantly characterizes human malaria infection and the production of proinflammatory cytokines such as interferon gamma (IFN-*γ*), IL-6, tumor necrosis factor (TNF-*α*), and other inflammatory cytokines [20, 21]. These inflammatory cytokines are considered critical for the resolution of parasitemia and control of malaria infection, especially during the early stages of* P. falciparum* infection [22, 23]. Conversely, if these robust inflammatory responses are not regulated during chronic malaria infection, they can lead to immunopathology and severe forms of the disease response [24–26]. Regulatory cytokines, including interleukin (IL)-10 and transforming growth factor beta (TGF-*β*), were shown to have an important role as an immunoregulator of the infection caused by* P. falciparum* in neutralizing the effects of Th1 inflammatory responses associated with immune pathology and the more severe forms of* P. falciparum* infection [24]. There also a range of other mediators, such as IL-4 and nitric oxide, that have been linked to disease severity in malaria-infected individuals [27, 28].

On the other hand, helminths infections have a profound effect on the immune system, resulting in a strong immunoregulatory and Th2 response that can inhibit the ability of the host to mount a Th1 type immune response. Indeed,* Ascaris* sp. infection induces anti-inflammatory Th2 responses [29–31] and is also associated with an immunoregulatory immune response, defined by elevated levels of interleukin (IL)-10 and transforming growth factor-*β* (TGF-*β*) [32, 33]. Therefore, it is expected that the influence of helminthic infection on the immune system could extend to the immune response against malaria due to the anti-inflammatory effect of cytokines induced by helminthes and thereby possibly affect the course of malaria infection [12, 34, 35].

Due to the recognized ability of some helminths to elicit anti-inflammatory cytokines and that many parasites manipulate host cytokine pathways for their own benefit [36], we hypothesized that there may be a counterbalance between proinflammatory cytokines, associated to malaria, and anti-inflammatory cytokines, associated to helminths or protozoa intestinal parasites. Although cytokine responses have been extensively described in* P. falciparum* infection, few studies have looked at systemic cytokine concentration in coinfection of intestinal parasites and malaria. We believe that comprehensive profiling of serum levels of multiple inflammatory markers such as cytokines, chemokine, C-reactive protein, and nitric oxide (NO) would provide greater insight into their utility to differentiate infected individuals with concurrent malaria and intestinal parasites to individuals with either infection alone. In this regard, the ability to measure numerous molecules in a single sample and to visualize changes in inflammatory markers, including cytokine networks in single and malaria and intestinal parasites coinfected individuals is critical to advance our understanding of the immune response to pathogens. Therefore, we applied both traditional univariate and multivariate analysis to the data in order to identify the type of response that develops during coinfection and which inflammatory markers are important.

## 2. Material and Methods

### 2.1. Study Area and Study Design

We conducted a cross-sectional survey in a rural settlement community of Porto Velho, municipality of Rondonia State, Brazilian Amazon. The settlement called Joana D'Arc is located 120 km of Porto Velho and the Brazilian Government in 2001 created it in order to give land to people. Joana D'Arc settlers were mostly rain forest native migrants, some with previous agricultural experience, but most with no knowledge of agricultural potential or techniques necessary for farming in a tropical rain forest area. They were low-income people attracted by free land and promised government support. Despite being a place where people lived for over 10 years, there is no health infrastructure in the area and the source of income of residents is from agriculture and small livestock to manufacture cheese. Samples and survey data were collected in 2010 and 2011, during the dry months of June-August, coinciding with the period of increased malaria transmission in Rondonia State. In order to be included in the study, participants had to meet the following criteria: (1) have been resident in the study area; (2) provided a stool samples; and (3) have given a blood sample for the collection of plasma and malaria diagnosis. A total of 264 participants met these criteria and formed our study population.

### 2.2. Sample Size

The sample size was estimated to determine prevalence of malaria using the formula for estimating single proportion at 95% confidence interval. The prevalence of coinfection is unknown in the area, so we used 0.50, to maximize sample size. Based on these entities and expected margin of error to be 0.1, 264 subjects were included in our study. All epidemiological, hematological, and cytokine quantification results were stored in Epi-Info version 3.2. Prior to analysis, the data were centered and standardized to ensure equal contribution of each parameter and to avoid differences due to scale.

### 2.3. Collection and Examination of Blood

The study team visited houses selected randomly to invite participation. After written informed consent and an epidemiological survey from all adult donors or from parents of donors in the case of minors, blood was drawn by venipuncture. At the time of blood sampling, thick and thin blood smears were performed and stained with 10% Giemsa to examine for the presence of malaria parasites. Parasitological evaluation was done by examination of 200 fields at 1000x magnification under oil-immersion. The parasitemia was expressed as the number of parasites/*μ*L of blood in the thick blood smear. Using the oil immersion objective, 500 leucocytes were counted at the same time as the number of parasites. Then, the number of parasites/*μ*L of blood was calculated by multiplying the number of parasites counted against 500 leucocytes and the number of leukocytes of the subject and dividing the product by 500. A researcher expert in malaria diagnosis examined all slides. To confirm the parasitological diagnosis, we performed molecular analyses of all samples using primers specific for genus (*Plasmodium* sp.) and species (*P. falciparum* and* P. vivax*). The amplification protocols were described previously by Snounou et al. [37]. Subjects were considered to have malaria if they were positive in the thick blood smear and/or PCR. Blood counts, including hematologic indices, were done using an automatic hematology analyzer (Pentra, Horiba Medical, Montpellier, France) and peripheral smears of blood samples were made for routine differential blood cellular quantification. A manual differential white blood cells count was also performed to distinguish the immature neutrophils.

### 2.4. Collection and Examination of Stool Samples

All individuals were requested to provide a morning fecal sample and a labeled screw-capped plastic container was provided. A single stool sample was collected from each subject on the following day and examined by a direct unstained wet smear in normal saline and Lugol's iodine solution at 100x and 400x by a technician with expertise in intestinal parasites identification. The physicians in our team provided medication after assessing the exams results of the participants and examining them. All participants found suffering from intestinal parasites infections were given complete treatment.

### 2.5. ELISA Specific for Protein C-Reactive

The CRP levels were determined in all plasma samples using an in-house ELISA. Microtiter plates (Nunc/MaxiSorp, Rochester, NY, USA) were coated with a goat anti-human-CRP antibody (Sigma, USA; catalogue C8284) in carbonate-bicarbonate buffer (TCO_4_) overnight at 4°C. The plates were then washed three times with phosphate-buffered saline-0.05% Tween 20 (PBST) and plasma samples diluted 1 : 500 in PBST were incubated with the plates for 1 h at 37°C. The plates were then washed three times and incubated with rabbit anti-human-CRP antibody (Sigma, USA; catalogue C3527) in PBST for 1 h at 37°C. The plates were washed three times and peroxidase-conjugated goat anti-rabbit-IgG antibodies (Sigma, USA; A0545) were added. The wells were thoroughly washed to remove all unbound horseradish peroxidase (HRP)-conjugated antibodies and an o-phenylendyamine substrate solution was added to each well. The enzyme (HRP) and substrate were allowed to react for a short incubation period. The enzyme-substrate reaction was terminated by the addition of 2 N H_2_SO_4 _and the degree of color change was measured at 492 nm ± 2 nm in a spectrophotometer (SpectraMax 250; Molecular Devices, Sunnyvale, CA). The plasma concentration of CRP was determined by comparison to standard concentrations of purified human CRP (Sigma, St. Louis, USA). The range of detection of CRP was 0.01–320 *μ*g/mL. Sera from noninfected individuals were used on every plate as negative controls. Specific CRP optical density values were converted to concentration values (*μ*g/mL) using sigmoidal curve-fit equations derived from CRP standard curves.

### 2.6. Griess Microassay Detection of Nitrite and Nitrate

A modified Griess reaction was used to detect nitrite and nitrate ([38], modified by [39]). The NO levels in samples were indirectly measured after first converting nitrates to nitrites with a nitrate reductase treatment (*Aspergillus* species NAD [P] H, Sigma, UK) and NADPH *β*-nicotinamide adenine dinucleotide phosphate (Sigma Diagnostics, St. Louis, USA). Griess reagent [5% phosphoric acid, 1% sulphanilic acid, and 0.1% N-(1-naphthyl-1)-ethylendiamine dihydrochloride, all from Sigma, UK, dissolved in 100 mL deionized water] was added and proteins were subsequently precipitated by trichloroacetic acid (BDH, England). The tube contents were mixed and centrifuged (Eppendorf centrifuge 5415 C, Germany); two samples of each supernatant were transferred to a flat-bottomed microplate and their absorbencies were read at 520 nm using a microplate reader (SpectraMax, Molecular Devices Inc). NO values were calculated from standard calibration plots [39].

### 2.7. Multiplex Microsphere Cytokine Immunoassay

Cytokine concentrations in plasma samples were determined using high throughput magnetic bead-based BioPlex assay. Thirteen cytokines IL-1*β*, IL-2, IL-4, IL-5, IL-6, IL-7, IL-10, IL-12 p70, IL-17A, IFN-*γ*, TNF-*α*, G-CSF, and granulocyte-macrophage colony-stimulating factor (GM-CSF) ] and three chemokines (IL-8, MCP-1, and MIP-1*β*) were analyzed using a BioPlex Kit assay (Bio-Rad Laboratories, Hercules, CA, USA) following the manufacturer instructions as described in [40]. Briefly, 50 *μ*L of standard or test sample along with 50 *μ*L of mixed beads was added into the wells of a prewetted 96-well microtiter plate. After 1 h of incubation and washing, 25 *μ*L of detection antibody mixture was added and the samples were incubated for 30°C min and then washed. Finally, 50 *μ*L of streptavidin-PE was added and, after 10°C min of incubation and washing, the beads were ressuspended in 125 *μ*L assay buffer and analyzed using a BioPlex suspension array system (Bio-Rad Laboratories) and the BioPlex manager software (v.3.0). A minimum of 100 beads per region were analyzed. A curve fit was applied to each standard curve according to the manufacturer's manual and sample concentrations were interpolated from the standard curves. The lower limits of cytokines detection using this method were MIP-1*β*, 1.69 pg/mL; IL-6 1.25 pg/mL; IFN-*γ*, 0.88 pg/mL; IL-5, 0.84 pg/mL; GM-CSF, 0.47 pg/mL; TNF-*α*, 0.82 pg/mL; IL-2, 0.29 pg/mL; IL-1*β*, 0.73 pg/mL; IL-13, 1.1 pg/mL; IL-4, 0.78 pg/mL; MCP-1, 1.64 pg/mL; IL-8, 1.01 pg/mL; IL-10, 0.4 pg/mL; G-CSF, 1.89 pg/mL; IL-7, 1.1 pg/mL; IL-12p70, 0.57 pg/mL; and IL-17A, 0.38 pg/mL TNF-*α*, 0.10 pg/mL. For all samples, the quantification of the analytes was done in a single day to avoid freeze-thaw cycles.

### 2.8. Statistical Analysis

We compared epidemiological and hematological parameters and cytokine production between groups, using a permutation-based ANOVA (999 permutations) followed by a post hoc test (Tukey HSD), to test for pairwise differences. Permutation-based ANOVA generates the null distributions from the data, avoiding the problems related to the violation of normality and homogeneity [41]. We also used Chi-squared test to determine the significant differences between proportions in binary variables (sex). A principal component analysis (PCA) was applied to the dataset containing cytokines, chemokines, and inflammation markers, to detect the variation patterns related to the studied groups and to identify variables accounting for the majority of the variation within the dataset. PCA is a widely used ordination methodology that reduces dimensionality of multivariate data and detects variables that are more important to explain the variance structure of the data, generating orthogonal (independent) axes using linear combinations of the original variables. PCA can be interpreted numerically: each axis (principal component, PC) is described by an eigenvalue related to the amount of variation that it explains, so that the first PC will always explain more variation than the second, and so on. In addition, variables and units have coordinates (“loadings”) along these PCs, which indicate their contribution to each PC. PCA can be also interpreted visually, from the origin of the graph: variables cytokines, chemokines, CRP, and NO (inflammatory mediators) and experimental units (patients) will be located according to their correlations, and distance from the origin means higher contributions to the overall variation (higher absolute loadings). Angles from the origin are roughly proportional to correlation: collinear vectors (approaching 0° or 180°) can be interpreted as positively or negatively correlated and right angles indicate independence (orthogonality).

To further investigate the relationship between infection groups and cytokine profile, we elaborated a heatmap that relates two hierarchical cluster analyses in a bidimensional plot.* Z*-scores were calculated from transformed values of cytokine levels and represent standard deviations from the population mean:* Z*-score = [(individual cytokine values − population cytokine mean value)/population cytokine standard deviation]. Cluster analyses were performed from* Z*-scores using Euclidean distance metrics and Ward as the linkage algorithm. All analyses were performed using* R* statistical environment [42]. Permutation ANOVA was performed with* lmPerm* package [43] and PCA analysis with* vegan* [44] and* gplots* package [45] was used to build the heatmaps.

### 2.9. Ethical Consideration

The study was conducted after obtaining ethical clearance from Fundação Oswaldo Cruz Ethical Committee (CEP/FIOCRUZ, 492/08). Individual oral and written consents were obtained from all participants. Donors positive for* P. vivax* and/or* P. falciparum* at the time of blood collection were subsequently treated using the chemotherapeutic regimen recommended by the Brazilian Ministry of Health.

## 3. Results

### 3.1. Malaria and Intestinal Parasites Infections

**Figure 1:**
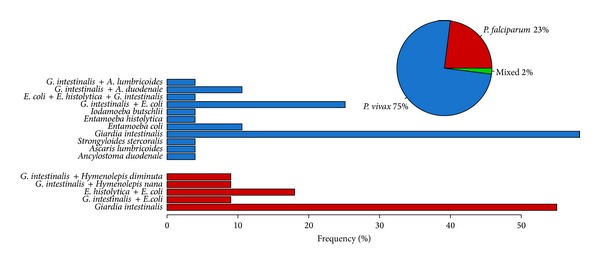
Frequency of individuals infected with* P. vivax* (blue) and* P. falciparum* (red) in the population of study. In the barplot the specific frequency of each species of intestinal parasites discriminated by* P. vivax* and* P. falciparum* is represented.

Of the 353 individuals analyzed at baseline, 264 subjects were included in our study, 16 (6.1%) were infected with malaria only, 48 (18.2%) were coinfected with malaria and intestinal parasites, 98 (37%) were infected with intestinal parasites only, and 102 (38.7%) were uninfected with either malaria or intestinal parasites.* P. vivax* was more prevalent in both malaria infected only (81.2%) and coinfected with intestinal parasites (75%). Protozoan parasites comprised the bulk of the infections in subjects infected with intestinal parasites only (70.4%) or coinfected with malaria and intestinal parasites (81.2%). The prevalence of intestinal parasites was significantly higher in individuals infected with malaria than with those who were not infected (adjusted OR = 3.1, 95% CI = 1.66–5.86 *P* = 0.0003). The most prevalent protozoan was* Giardia intestinalis* and helminths were* Ancylostoma duodenale* and* Strongyloides stercoralis*. Multiple protozoa species were common in both intestinal parasites only (14.5%) and coinfected with malaria (33.3%) while only one subject presented multiple helminthes species infection. Dual, triple, and quadruple protozoan and helminthes infections were observed. Data in Table 1 summarizes the prevalence of single and multiple parasites species infections. In coinfected subjects, the prevalence of* P. vivax* malaria was 75% (36) and* P. falciparum* was 23% (11) whereas 2% (1) had mixed species infection.* G. intestinalis* was the most prevalent intestinal parasite in both* P. falcilparum* and* P. vivax* coinfections. However, in* P. vivax* coinfected subjects the species of protozoans and helminthes were more diverse (Figure 1).

### 3.2. Infection Groups, Epidemiological, and Hematological Data

As shown in Table 2, the infection groups were defined by the presence or absence of malaria infection and/or intestinal parasites infection, resulting in the following groups: malaria (M), coinfected (CI), intestinal parasites (IP), and uninfected (UN). The majority of the subjects infected with malaria was male and presented general clinical malaria symptoms such as history of fever and headache. We did not observe differences in parasitemia between Malaria and coinfected groups. There were no differences in age, years of residence in endemic area (TR), years of residence in Rondonia (TRO), and months since last malaria episode (LME). However the number of past malaria episodes (PME) was higher in the groups negative for malaria when compared with malaria group (intestinal parasite *P* < 0.0001 and uninfected *P* < 0.05). The analysis of some hematological parameters revealed that malaria and coinfected groups were similar and no differences were observed in the median hemoglobin, platelets, lymphocytes, band cells values, and* Plasmodium* parasites counts. However, they differ from intestinal parasites and uninfected groups presenting lower mean lymphocytes and platelets counts while band cells counts were higher. Hemoglobin levels in malaria and coinfected groups were slightly higher when compared with the uninfected group.

### 3.3. Levels of Inflammatory Mediators

**Figure 2:**
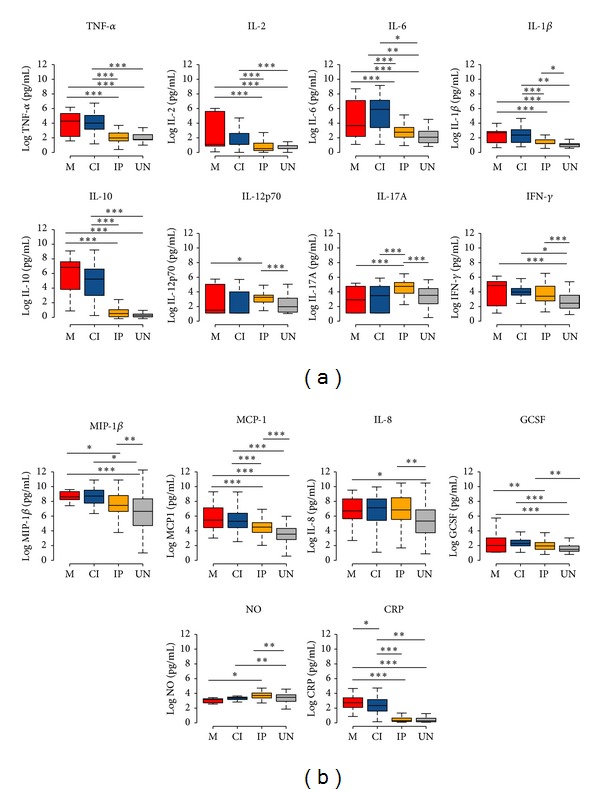
Boxplots of the levels of (a) cytokines, (b) chemokines, and inflammation markers in the groups (M = red, CI = blue, IP = orange, and UN = gray). Differences were calculated using a TukeyHSD from a permutation ANOVA over cytokine values transformed with Log. Significant statistical differences are represented in the bars and the level of significance expressed as ∗∗∗*P* < 0.0001, ∗∗*P* < 0.001, and ∗*P* < 0.05.

Firstly, we analyzed the median plasma levels of individual cytokines, chemokines, NO, and CRP comparing the concentration between groups (Figure 2). Cytokines levels varied widely among the cytokines ranging from 0.3 pg to 6952 pg. The cytokines IL-5, IL-7, and GM-CSF were low in most plasma samples (data not shown). Most remarkably, Malaria group presented the highest levels of CRP and intestinal parasites the highest level of IL-12p70, IL-17A, and NO. In both malaria and coinfected groups the median levels of TNF-*α*, IL-2, IL-10, IL1-*β*, MCP-1, and IL-6 were observed to be mostly significantly increased compared with those intestinal parasites and uninfected groups (*P* < 0.001 for all comparisons). The median levels of IFN-*γ* and IL-8 were increased in all groups compared to uninfected group. Changes in cytokine and chemokine levels were very similar in malaria and coinfected groups and with this analysis we could not associate changes in cytokine profile that could be due to coinfection.

### 3.4. Principal Component Analysis (PCA) and Cluster Analysis of Inflammatory Mediators

**Figure 3:**
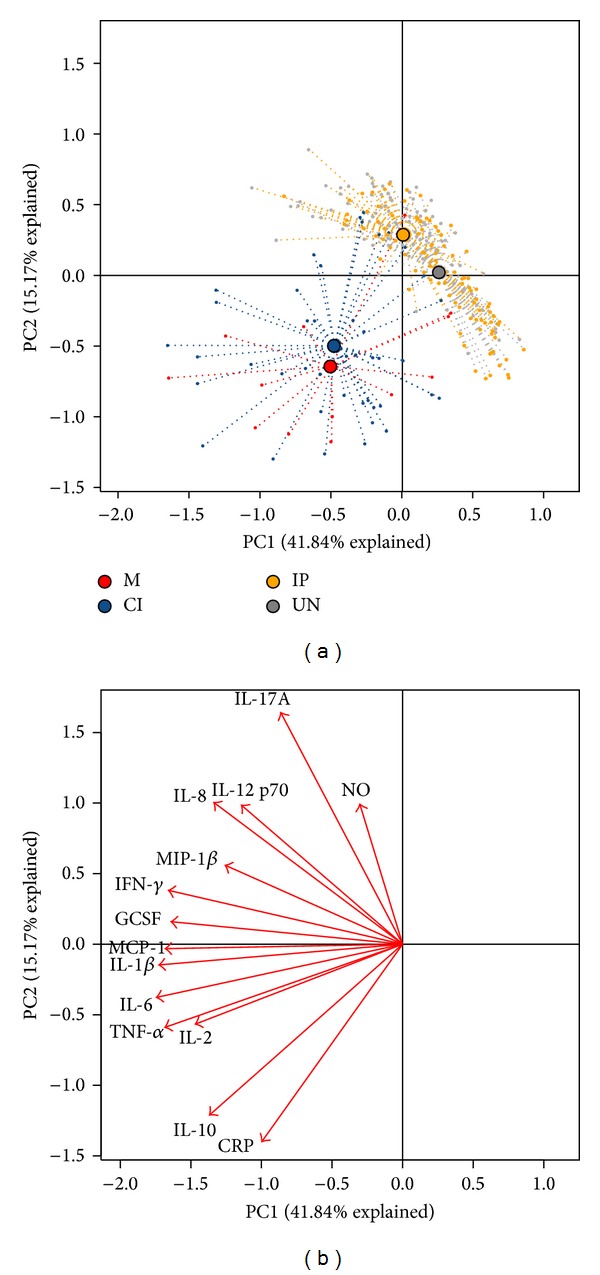
Principal component analysis (PCA) of inflammatory mediators (cytokine, chemokines, CRP, and NO). (a) Results are shown for M, CI, IP, and UN groups. Each point represents an individual from a group and each group has a color code: M = red, CI = orange, IP = blue, and UN = gray. (b) Arrows indicating the direction of maximum change while the length of arrows represents the magnitude of the change. The explanation of the first principal component (PC1) explained 41.84% of the variation of the data and the second principal component (PC2) explained 15.17%. The separation of CI and M individuals from IP and EXP and the association of some cytokines with the different groups can be observed.

**Figure 4:**
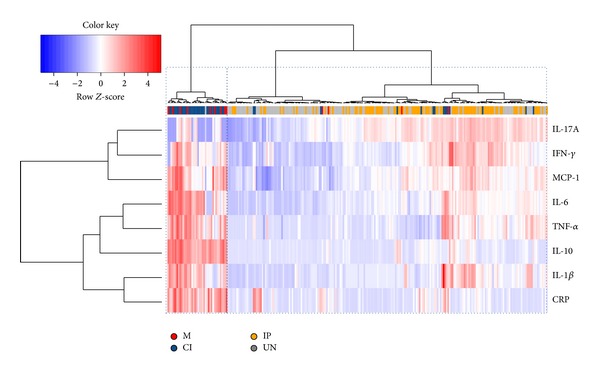
Two-way coupled cluster analysis (heatmap). Each cell indicates the value of a single mediator of inflammation among the studied individuals. The horizontal cluster illustrates the grouping of individuals considering seventeen cytokines, C-reactive protein, and nitric oxide. The vertical cluster shows the grouping of mediator of inflammation such as cytokines meaning that cytokines that presented more similar responses are closer in the clusters. The red color in the cells indicates high values and the blue color indicates low values in the production of cytokines and inflammatory proteins. The white color indicates no changes in the cytokine levels. The colors under the horizontal cluster represent the groups (M = red, CI = orange, IP = blue, and UN = gray) of each individual. Sample clustering resulting from the algorithm applied is shown at the top of the graph as a horizontal dendrogram, with an indication of the group to which each individual sample belongs. The horizontal-dotted boxes show the clusters of individuals obtained. The first vertical box with dotted lines represents the group of individuals with malaria only (M) and malaria-intestinal parasites coinfected (CI). The second box shows the uninfected (UN) and IP individuals.

In order to assess differences in the whole multivariate set of cytokines, chemokines, NO, and CRP data in individuals between the groups, we performed an exploratory principal component analysis (PCA), a multivariate technique to identify whether inflammatory mediators could indicate coinfection.  Figure 3(a) displays a PCA of inflammatory mediator for the groups M, CI, IP, and UN. The dotted line connects each individual to the centroid of its group and the position of the centroids indicates that there was an overall difference in the mediators between two groups: M and CI and IP and UN groups. In these two groups, the individuals were gathered by their similar inflammatory mediator's profiles. The differences in the inflammatory mediators between M and CI groups from IP and UN groups were higher levels of IL-1*β*, IL-6, TNF-*α* IL-10, and CRP and decreased levels of IL-17A and NO while IP and UN presented higher levels of IL-17A and NO and decreased levels of IL-10 and CRP (Figure 3(b)). In order to characterize cytokine inflammatory mediators in each studied individual, we applied a two-dimensional clustering analysis (Figure 4) using the cytokines that presented the largest loading values in the PCA analysis (IL-6, IL-1*β*, TNF-*α*, MCP1, IFN-*γ*, IL-17A, CRP, and IL-10). This method allows coupled clustering of both the subject and the measured parameters without taking in account the groups. Interestingly, the clustering algorithm could discriminate two major clusters: one cluster included increased levels of IL-6, TNF-*α*, IL-10, and CRP and low levels of IL-17A predominantly in individuals from M and CI groups and the second cluster included low levels of almost all inflammatory mediators predominantly in individuals from UN group and increased levels of IL-17A predominantly in individuals from IP group.

## 4. Discussion

Overlapping distribution of intestinal parasites and malaria might result in high rate of coinfection. In the studied population 24% of the individuals were infected with* Plasmodium,* 55% with intestinal parasites, and 18% with malaria and intestinal parasites. In the studied area, the likelihood of being infected with malaria was significantly higher in individuals infected with intestinal parasites. Several studies from human populations conducted in Africa reveal that helminthic infection can have a negative effect on host response to malaria, including increased susceptibility to* Plasmodium* infection and increased severity of disease [11–14]. In our study, although malaria was more frequent in individuals infected with intestinal parasites, hematological parameters and parasitemia did not differ between coinfected and malaria single infected individuals. In both groups anemia was not frequent and changes in lymphocytes, and platelets and band cells seem to be due to acute malaria infections while eosinophil levels were high only in intestinal parasites group. Although anemia and thrombocytopenia are the most prominent alterations in acute malaria infection and in coinfections with helminths, hematological changes in these infections are a wide and contradictory event [40, 46–50].

Differences in results obtained in different studies might depend on the species of the intestinal parasites and the age of studied population. While most of the coinfection studies are with helminthes and in children [1, 10], in our study the most prevalent intestinal parasites in the population were protozoans and the participants were adults. In addition, our sample size may be small and could not allow the stratification of the intestinal infections by helminthes and protozoans, a factor which may account for these differences.

The influence of intestinal parasites (mainly helminthes) in* Plasmodium* coinfection has gained interest because it has been hypothesized that Th2 polarized immune response elicited by helminthes could alter the natural immune response of the host to* Plasmodium* parasites [12, 34, 36]. Most of the studies associate cytokine profile mainly with the immunopathology of severe/complicated malaria [24, 51]. Few studies looked at systemic cytokines concentration in coinfection comparing plasma of infected individuals with concurrent malaria and intestinal parasites with either infection alone [52–55]. To our knowledge, this is the first study that evaluates 16 cytokines, CRP, and NO in malaria coinfection with intestinal parasites. In our study, the analysis of individual cytokines, chemokines, CRP, and NO could not detect changes associated to coinfection. However, the use of principal component analysis and cluster analysis provided evidence that groups of individuals with malaria (M and CI) could be discriminated from the groups of individuals negative for malaria (IP and UN) based on inflammatory mediators profile. They formed two separate groups based on the levels of cytokines, CRP, and NO. For malaria infected individuals (M and CI) the profile was high levels of IL-1*β*, IL-6, TNF-*α* IL-10, and CRP and decreased levels of IL-17A while for malaria negative individuals (IP and UN) the profile was high levels of IL-17A, NO and decreased levels of IL-10 and CRP.

The high production of CRP, IL-10, TNF-*α*, and IL-6 observed in our analysis in M and CI groups is reported in several studies in acute malaria infections [50, 56, 57]. In Brazilian populations, IL-10 and CRP are an important marker of acute malaria caused by* P. vivax* [40, 50, 56].

The role of cytokines production in acute malaria is far from being understood and little is known about their effect in coinfection with other parasites. In our study, the levels of inflammatory mediators in individuals with acute malaria did not differ from individuals coinfected with intestinal parasites when compared with individuals single infected with malaria. However, few reports demonstrated altered cytokines levels in children and adults coinfected with* P. falciparum* and* Schistosoma haematobium*. Higher IFN-*γ* and similar TNF-*α*, TGF-*β*, and IL-10 levels were found when comparing coinfected and single* P. falciparum* infected children [52] while, in adults, higher IFN-*γ*, TNF-*α*, and TGF-*β* levels were detected [53]. Elevated IL-6 and IL-10 were also associated with acute malaria in children but the levels were lower in children coinfected with* S. haematobium* when compared to children infected with* S. haematobium* alone [54]. In these studies the question of whether the high concentration of cytokines in coinfected individuals had a negative or positive effect on malaria was not addressed.

It is worth it to highlight that, specifically using PCA analysis, we observed the importance of the contribution of IL-17A to separate the M and CI from IP and UN, although IL-17A seems to be higher in IP individuals. It has been observed that IL-17 is an important marker of intestinal inflammation contributing to enhance innate barrier defenses at mucosal surfaces [58, 59]. Interestingly in the groups CI and M we also detected decreased levels of NO. This inflammatory marker has been described to be protective against* P. falciparum in vitro*, and low levels of this marker have been associated with suppression of NO synthesis depending of the gravity in malaria [27]. The contribution of intestinal parasites coinfection in the pathogenesis of malaria is still controversial, and its role is probably specific depending on the type of parasite involved in the coinfection. In our study in spite of the great amount of inflammatory markers evaluated, the use of multivariate analysis techniques proved to be an excellent tool to find hidden patterns in complex data systems including cytokine studies [57, 60–62]. Indeed, using cluster analysis, Prakash et al. [61] detected differences of plasmatic cytokines between individuals with mild, severe noncerebral, and cerebral malaria. In our study, this analysis was able to detect differences between individuals with malaria versus without malaria but was not able to detect differences between malaria versus intestinal parasites coinfected individuals. Therefore, it seems that intestinal parasites coinfection (mainly protozoan) does influence in the plasmatic cytokine levels of individuals with acute malaria and the real influence of these infections could be perceived in regions highly endemic for specific parasites such as* Ascaris lumbricoides* and* Schistosoma mansoni*.

## 5. Conclusion

In conclusion, our data suggest that, in our population, the infection with intestinal parasites (mainly protozoan) does not modify the pattern of cytokine production in individuals infected with* Plasmodium*.

## Figures and Tables

**Table 1:** Prevalence of malaria and intestinal parasites in the studied population.

	Number of cases	Prevalence (%)
Infected with malaria only		
*P. vivax *	**13**	**81.2**
*P. falciparum *	**3**	**18.8**

Total	**16**	**6.1**

Infected with intestinal parasites only		
Protozoa	**69**	**70.4**
*Giardia intestinalis *	48	69.6
*Entamoeba coli *	7	10.1
*Entamoeba histolytica *	3	4.3
*Iodamoeba butschlii *	1	1.5
*G. intestinalis * + (*I. butschlii *or *E. histolytica* or* E. coli*)	3	4.3
*E. histolytica * + (*I. butschlii *or *E. coli*)	2	2.9
*E. coli * + *E. histolytica * + *G. intestinalis *	3	4.3
*E. coli * + *E. histolytica * + *I. butschlii *	1	1.5
*E. coli * + *E. histolytica * + *G. intestinalis* + *I. butschlii *	*1 *	1.5
Helminths	**17**	**17.4**
*Ancylostoma duodenale *	6	35.3
*Strongyloides stercoralis *	5	29.4
*Ascaris lumbricoides *	3	17.6
*Trichuris trichiura *	2	11.8
*S. stercoralis * + *T. trichiura *	1	5.9
Protozoa + Helminthes	**12**	**12.2**
*E. coli* + *A. lumbricoides *	2	16.7
*G. intestinalis * + (*A. duodenale* or *T. trichiura* or *S. stercoralis*)	7	58.4
*G. intestinalis * + *I. butschlii * + *A. lumbricoides *	1	8.3
*E. coli * + *E. histolytica * + *A. duodenale *	1	8.3
*E. coli * + *E. histolytica * + *I. butschlii * + *S. stercoralis *	1	8.3

Total	**98**	**37.1**

Coinfected with malaria + intestinal parasites		
Malaria + Protozoa	**39**	**81.2**
*G. intestinalis *	23	59.0
*E. coli *	3	7.7
*E. histolytica* or *I. butschlii *	2	5.2
*E. coli * + (*G. intestinalis* or *E. histolytica*)	10	25.6
*E. coli * + *E. histolytica * + *G. intestinalis *	1	2.6
Malaria + Helminths	***3***	***6.3***
*A. duodenale* or *A. lumbricóides* or *S. stercoralis *	3	100
Malaria + Protozoa + helminthes	*6 *	*12.5 *
*G. intestinalis* + (*A. duodenale* or *A. lumbricoides*)	4	66.7
*G. intestinalis* + (*Hymenolepis nana* or *Hymenolepis diminuta*)	2	33.3

Total	**48**	**18.2**

Uninfected	**102**	**38.6**

**Table 2:** Epidemiological and hematological data in the studied groups.

	Infection groups			
	Malaria	Coinfected	Intestinal parasites^a^	Uninfected^b^
	*N* = 16 *n* (%)	*N* = 48 *n* (%)	*N* = 98 *n* (%)	*N* = 102 *n* (%)
Gender *n* (%)				
M	11 (69)	34 (71)	49 (50)	48 (47)
F	5 (31)	14 (29)	49 (50)	54 (53)
Age	24 (21–33)	31 (22–41)	30 (14–43)	29 (14–38)
TR	22 (16–27)	23 (18–32)	23 (14–34)	24 (13–31)
TRO	21 (10–24)	22 (15–27)	18 (11–30)	24 (13–30)
LME	6 (0–66)	3 (0–16)	24 (6–60)	10 (1–36)
PME	5 (2–8)^a***b*^	4 (1–10)^a*^	4 (2–60)^b*^	5 (2–14)
Hematological				
Lymphocytes (mm^3^)	1316 (863–1982)^b***^	1170 (789–1826)^a***b***^	2178 (1813–2725)	2068 (1697–2467)
Platelets (mm^3^)	166 (148–204)^a*b***^	152 (106–197)^a***b***^	214 (173–245)^b*^	233 (193–286)
Band cells (mm^3^)	34 (0–141)^a***b***^	26 (0–143)^a***b***^	0 (0-0)	0 (0-0)
Eosinophils (mm^3^)	73 (36.75–138.75)^a*^	104 (42.5–328.5)^a*^	328 (185–720)^b*^	245 (146.75–484.00)
Hemoglobin (g/dL)	12.7 (12–14)^b*^	13.2 (12.2–14)^b*^	13.8 (12.8–15)	13.8 (13–14.7)
Parasitemia (parasites/*μ*L)	2740 (738–5591)	1816 (641–5700)	—	—

*n* (%): number of samples (percentage); TR: Years of residence in endemic area; TRO: Years of residence in Rondonia; LME: Months since last malaria episode and PME: number of past malaria episodes. The variables Age, TR, TRO, LME and PME, values are expressed as Median (25%–75%). Differences were calculated using a TukeyHSD from a permutation based ANOVA. Differences of parasitemia between Coinfected and Malaria group were calculated using a permutation *t* test. ^a^differences between indicated group and Intestinal parasites; ^b^differences between indicated group and Uninfected. Statistical differences of epidemiological parameters were expressed as ***P < 0.0001, **P < 0.001, *P < 0.05.
